# Population delimitation across contrasting evolutionary clines in deer mice (*Peromyscus maniculatus*)

**DOI:** 10.1002/ece3.3

**Published:** 2011-09

**Authors:** D-S Yang, G Kenagy

**Affiliations:** Burke Museum and Department of Biology, Box 351800, University of WashingtonSeattle, Washington 98195, USA

**Keywords:** Gene flow, genealogical discordance, local adaptation, *Peromyscus maniculatus*, population delimitation

## Abstract

Despite current interest in population genetics, a concrete definition of a “population” remains elusive. Multiple ecologically and evolutionarily based definitions of population are in current use, which focus, respectively, on demographic and genetic interactions. Accurate population delimitation is crucial for not only evolutionary and ecological population biology, but also for conservation of threatened populations. Along the Pacific Coast of North America, two contrasting patterns of geographic variation in deer mice (*Peromyscus maniculatus*) converge within the state of Oregon. Populations of these mice diverge morphologically across an east–west axis, and they diverge in mitochondrial DNA haplotypes across a north–south axis. In this study, we investigate these geographically contrasting patterns of differentiation in the context of ecological and evolutionary definitions (paradigms) of populations. We investigate these patterns using a new and geographically expansive sample that integrates data on morphology, mitochondrial DNA, and nuclear DNA. We found no evidence of nuclear genetic differentiation between the morphologically and mitochondrially distinct populations, thus indicating the occurrence of gene flow across Oregon. Under the evolutionary paradigm, Oregon populations can be considered a single population, whereas morphological and mitochondrial differentiations do not indicate distinct populations. In contrast, under the ecological paradigm morphological differentiation indicates distinct populations based on the low likelihood of demographic interactions between geographically distant individuals. The two sympatric but mitochondrially distinct haplogroups form a single population under the ecological paradigm. Hence, we find that the difference between evolutionary and ecological paradigms is the time-scale of interest, and we believe that the more chronologically inclusive evolutionary paradigm may be preferable except in cases where only a single generation is of interest.

## Introduction

Populations are the fundamental unit of evolution, the scale at which the microevolutionary forces of natural selection, genetic drift, gene flow, and mutation act. Population delimitation is the analytical process by which researchers attempt to define boundaries between distinct populations. The current emphasis of conservation biology on conserving “evolutionarily significant units” (Ryder 1986, Moritz 1994, Hoglund 2009) relies on accurate designation of populations in order to apply conservation efforts effectively. Similarly, basic research on the ecology and evolution of wild populations also relies on accurate population delimitation. Thus, defining population boundaries is the crucial step for understanding the biology of all populations. Nevertheless, a concrete definition of “populations” remains elusive (Waples and Gagiotti 2006, Lowe and Allendorf 2010). Waples and Gagiotti (2006) address this problem by defining populations under separate “paradigms.”

In the ecological paradigm, the cohesive forces are largely demographic, and emphasis is on co-occurrence in space and time so that individuals have an opportunity to interact demographically (competition, social, and behavioral interactions, etc.). In the evolutionary paradigm, the cohesive forces are primarily genetic, and emphasis is on reproductive interactions between individuals.

Here, we investigate the applicability of these paradigms to populations of *Peromyscus maniculatus* (deer mice) in western North America, within the state of Oregon. We investigate these mice with methods that have commonly been used to delimit populations: analyses of mitochondrial DNA (mtDNA) and morphological differentiation. We then further compare our findings to results from nuclear DNA analyses, which are becoming more common for investigating population delimitation.

**Figure 1:**
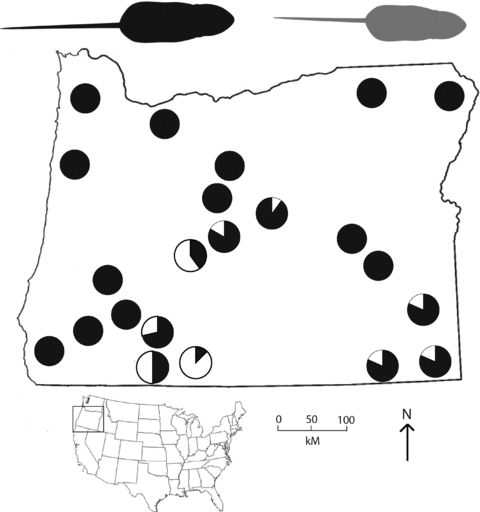
Axes of differentiation in deer mice (*P. maniculatus*) across the state of Oregon. Morphological variation shows a west–east axis of differentiation, with large, dark-colored, long-tailed mice in the west and small, light-colored, short-tailed mice in the east. Differentiation according to two major mitochondrial DNA clades is depicted for 22 localities with pie charts indicating the relative number of individuals belonging to Pacific Northwest (black) and Californian (white) clades (redrawn from Yang & Kenagy 2009). In general, the frequency of Pacific Northwest haplotypes increases from south to north.

Oregon populations of *P. maniculatus* represent an interesting conundrum for population delimitation. Evidence from field surveys and classical taxonomy indicates that the morphologies of Oregon *P. maniculatus* populations diverge across a west–east axis (Bailey 1936; Verts and Carraway 1998), where mice in the western coastal forests are dark-colored, large-bodied, and long-tailed and mice in the eastern interior sagebrush-steppe are light-colored, small-bodied, and short-tailed. Over the same geographic space, a north–south oriented contact zone also exists between two distinct western North American mtDNA clades of *P. maniculatus* (Yang and Kenagy 2009; Fig. 1). Thus, population delimitation based on morphology suggests distinct populations in eastern and western Oregon, whereas population delimitation based on mtDNA would indicate separate northern and southern populations.

Both mtDNA and morphological data have drawbacks when used for population delimitation. Morphology is subject to phenotypic plasticity (gene × environment interactions), which may indicate a misleading degree of population connectivity or isolation. Mitochondrial DNA may indicate population isolation despite interbreeding between populations because of the maternal inheritance of mtDNA, especially in species with male-biased dispersal such as *P. maniculatus* (King 1968). In contrast, nuclear DNA cannot respond plastically to environment within an individual's lifetime (unlike morphology) and reflect the genetic contribution of both parents (unlike mtDNA), thus making nuclear DNA data the most reliable method of detecting interbreeding and connectivity between populations.

In this study, we test the validity of population designations based on morphological and mtDNA data under the above ecological and evolutionary “population” paradigms. We conduct replicate sampling transects across both the morphological and mtDNA axes of differentiation. We present statistically rigorous morphological data from populations to determine the validity of the previously reported west–east/forest-sagebrush axis of morphological differentiation. We provide new mtDNA data along the new sampling transects to expand the geographic sampling of admixture between northern and southern mtDNA haplotypes within Oregon. We then explore whether morphological and mtDNA differentiation corresponds to nuclear DNA differentiation. Next, we use these nuclear genetic data to test for genetic connectivity across Oregon populations to determine whether separate “evolutionary” populations exist. Finally, we interpret the results under the both population paradigms to determine whether the populations delimited under each paradigm are congruent.

## Methods

### Sampling

**Figure 2:**
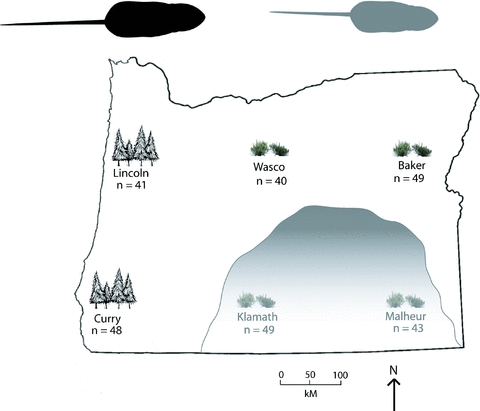
Map of six localities sampled for the current study, transecting the morphological and mtDNA differentiation gradients shown in Figure 1. The two westernmost localities are associated with wet coastal forest and contain large, dark-colored, long-tailed mice. The four interior localities are associated with sagebrush-steppe and contain small, light-colored, short-tailed mice. Sample size for each locality is given below the locality name (county). The geographic position of the observed mtDNA contact zone (Yang & Kenagy (2009) is indicated by the shaded overlay.

We sampled 270 *P. maniculatus* from six localities across Oregon (Fig. 2). Thirty-six of these individuals were collected in summer 2006 and the remainder in summer 2007. The localities were arranged in a 2 × 3 grid across Oregon, with localities separated by about 250 km. We collected between 40 and 49 mice per locality (Appendix S1), with the goal of being able to accurately infer migration rates between each locality (Paetkau et al. 2004). All specimens, including frozen tissue samples, were deposited in the mammal collection of the Burke Museum (UWBM). Two of the localities (Lincoln and Curry Counties) are within the wet coastal forest, and four (Wasco, Klamath, Baker, and Malheur Counties) are within the interior sagebrush-steppe.

### Mitochondrial DNA data

We sequenced a portion of the mtDNA control region for eight randomly chosen adult individuals from each of the six localities (total *n* = 48, Appendix S1). We performed the amplification, sequencing, and alignment as in Yang and Kenagy (2009) with the exception that we sequenced the PCR products in both directions using TDKD (5′-CCTGAAGTAGGAACCAGATG-3′, Kocher et al. 1993) as the reverse primer. The data from these newly sequenced individuals were uploaded to GenBank (accessions JF451059 to JF451102).

### Nuclear sequence data

We chose to utilize nuclear sequence for comparison to the mtDNA data for the purpose of gaining additional information about population relationships by using a phylogenetic approach and observing branching events in the phylogeny, information that would not be apparent with the clustering method used for microsatellite data by Yang and Kenagy (2009). For the 48 individuals sampled for mtDNA, we used PCR to also amplify two putatively neutral nuclear loci using primers from Storz et al. (2007): β-Fibrinogen intron 7 (766 bp, Forward: 5′ATTCACAACGGCATGTTCTTCAG3′, Reverse: 5′AANGKCCACCCCAGTAGTATCTG3′); and RAG1 exon 1 (1261 bp, Forward: 5′TCCATGCTTCCCTACTGACCTG3′, Reverse: 5′TGGCTTCTGGTTATGGAGTGGA3′). We used these PCR products to subclone six copies of the gene per individual using the TOPO4 cloning kit (Invitrogen, Carlsbad, California). We then amplified these six subclones using a standard colony PCR protocol (total copies = 288 subclones per gene), and sequenced each subclone in both forward and reverse directions using the University of Washington High-Throughput sequencing service (htseq.org). Using Sequencher 4.1 (Gene Codes) we merged the forward and reverse sequence data for each subclone and removed redundant subclones for each individual. Finally, we aligned the data set for each gene by eye. The sequence data for each clone were uploaded to Genbank for each locus (β-Fibrinogen Intron 7: Accession HM072088-72183, RAG1: Accession HM072184-42277)

### Phylogenetic inference of mitochondrial and nuclear sequence data

We used phylogenetic inference to identify which mtDNA haplogroup (Californian or non-Californian; Yang and Kenagy (2009)) each of the 48 selected individuals belonged to and to determine whether nuclear sequence variation reflected the population structure found in mtDNA. We integrated the newly collected mtDNA data into the mtDNA data set of Yang and Kenagy (*n* = 455; 2009) for a total *n* = 503 and used jModelTest to estimate the most likely model of sequence evolution and the model parameters for each locus using the Akaike information criterion. We evaluated seven models of evolution and four parameters (unequal base frequencies, proportion of invariable sites, and Γ- distribution of rate variation) per model (total number of models = 56). We used these models and parameter values to perform maximum-likelihood based phylogenetic inference with PHYML 3.0 (Guindon and Gascuel 2003). We tested nodal support of the trees by repeating the phylogenetic analysis for 100 bootstrap pseudo-replicates.

For comparison, we also inferred the mtDNA phylogenies in a Bayesian statistical framework using Parallel MrBayes 3.1.2 (Huelsenbeck and Ronquist 2001; Ronquist and Huelsenbeck 2003; Altekar et al. 2004) through the Cornell BioHPC (http://cbsuapps.tc.cornell.edu/mrbayes.aspx). Each run used six substitution categories and included a proportion of invariant sites and a Γ-distribution of rate variation. For each locus, each run consisted of 20 million generations, saving every 20,000th tree, with the first 250 saved trees discarded as a “burn-in.” Each run consisted of four chains with default priors other than the branch length prior, which we set to an unconstrained value of 0.50 because of the short branches expected in this population study. For each run, we used AWTY (Wilgenbusch et al. 2004; Nylander et al. 2008) to graphically examine output values and to determine when the analysis runs had achieved convergence and stationarity of parameter values (Appendix S2).

To observe whether significant nuclear genetic geographic structure existed between localities, we used the coalescent-based phylogenetic inference program *BEAST (Drummond & Rambaut 2007) to infer a locality-based phylogeny using data from the two nuclear genes (β-Fibrinogen, RAG1). We set the substitution model for each gene to GTR + I +Γ with six gamma categories. The branch rates were estimated relative to β-Fibrinogen data. Each Markov Chain-Monte Carlo (MCMC) chain was run for 10 million generations logging parameter values every 10,000 iterations. All other settings were default.

### Morphological data

For the 206 adult specimens collected, we measured up to 19 quantitative morphological traits, depending on the condition of the skeletal material. We omitted 64 juveniles from our total sample of 270, based on pelage and weight. Before preparation of the museum specimens, we measured total length, tail length, body length, hind foot length, ear length, and weight. After skeletal preparation, we measured greatest length of the skull, length of the cranium, breadth of the rostrum, breadth of the braincase, depth of the braincase, length of the mandibular tooth row, length of the humerus, length of the forefoot, length of the femur, length of the hind foot, sacral width, number of presacral vertebrae, and number of postsacral vertebrae. These data were tabulated, standardized within each trait, and imported into R (R Development Core Team 2008).

### Nuclear microsatellite data

We genotyped all 270 individuals using one microsatellite locus from Mullen et al. (2006) and eight microsatellite loci from Kelly et al. (2002) (Appendix S4). After optimizing PCR conditions, we electrophoresed the samples using an ABI 3100 sequence analyzer and scored the data manually using GENEMAPPER software (Applied Biosystems). We imported the data into GENALEX6 (Peakall and Smouse 2006) to calculate molecular genetic diversity indices and a triangular molecular genetic distance matrix. We then imported this molecular genetic distance matrix into R.

### Analysis of morphological and neutral genetic differentiation

To test the hypothesis of morphological population differentiation among Oregon mice, we ordinated the morphological data along their major axes of variation by principal components analysis (PCA) using the vegan package (Oksanen et al. 2008) in R. We then tested whether each axis explained significantly more variation than expected from random chance alone by comparing variation among the ordinated axes to a null distribution of variation among axes generated by the broken-stick distribution (Frontier 1976) and also against a null distribution generated by 1000 Markov Chain-Monte Carlo randomizations of trait values.

To test whether the nuclear microsatellite data were differentiated in concurrence with the morphological data, we ordinated the triangular molecular genetic distance matrix generated by GENALEX using Principal Co-ordinate Analysis (PCoA) in vegan. We set the number of axes to nine (*k* = 9) to correspond to the number of loci genotyped for this analysis. We tested each PCoA axis for statistical significance by comparison to the broken-stick distribution.

As a statistical test for a correlation between morphological and nuclear genetic variation, we performed a Mantel test (Mantel 1967). We generated a triangular morphological distance matrix using Bray–Curtis distance using the vegdist function in vegan. We then performed the Mantel test with the MASS package included in R using Pearson's product-moment correlation. We tested the significance of the correlation against a null distribution generated by 1000 permutations of the data.

### Estimation of migration rates between sample localities

To examine rates of gene flow and the amount of genetic connectivity between sampled localities, we used the coalescent-based software program LAMARC 2.0 (Kuhner 2006) to infer values of θ*m*, where θ is the population genetics parameter 4*N_e_*µ and *m* is the migration rate between populations. We performed the analyses separately for the nine-locus microsatellite data set and the two-locus nuclear sequence data set. Each analysis consisted of three replicates, each with 10 initial chains of 30,000 generations with a 20,000 generation burn-in and two final chains of 220,000 generations with a 20,000 generation burn-in. Each analysis used a Bayesian approach to search parameter space and began with the default prior values. By acquiring 95% confidence intervals for θ*m* estimates, we could detect when migration rates between localities were statistically significantly different from zero.

### Estimation of population number

To estimate the number of populations present among the six localities we sampled, we analyzed the microsatellite data from all individuals with the program STRUCTURE 2.3.3 (Pritchard et al. 2000; Falush et al. 2003, 2007; Hubisz et al. 2009). We performed 10 runs each for *K* = 1 to *K* = 6. Each run consisted of a 10,000 generation burn in followed by a 100,000 generation data collection run. We used sampling location as a prior for each run. Each run started with a lambda model with a value = 1.0. Following these runs, we calculated the mean value of Ln P(D) for each *k* across the 10 runs. We then calculated the ΔLn P(D) between each adjacent value of *K* to find where ΔLn P(D) was maximized to indicate the most likely number of populations in the sample (Evanno et al. 2005).

## Results

### Mitochondrial versus nuclear DNA sequence differentiation

**Figure 3:**
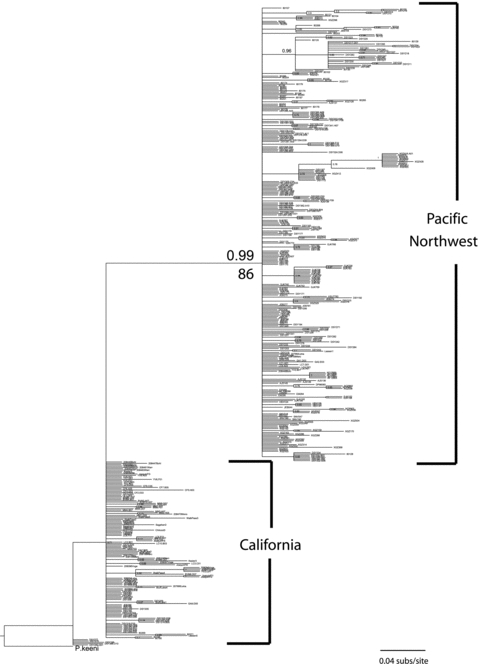
MtDNA tree from MR. BAYES analysis showing differentiation according to Pacific Northwest and Californian clades for deer mice across Western North America. Nodal support for the Pacific Northwest clade is shown above (Bayesian posterior probability) and below (maximum-likelihood bootstrap) the branch. Tree is rooted with a homologous Peromyscus keeni sequence (D-S. Yang, unpubl. data). The tree topologies inferred by maximum likelihood (not shown) and Bayesian inference methods were very similar.

**Figure 4:**
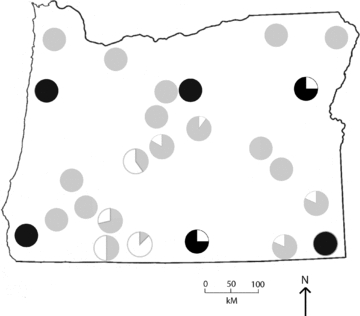
Relative frequencies of individuals belonging to Pacific Northwest (black) and Californian (white) clades among 48 individuals sampled at six localities for the current study, as displayed in Figure 1. Translucent pie charts represent data from Yang & Kenagy (2009), as shown in Figure 1. The new data indicate that the geographic distribution of Californian haplotypes in Oregon may not follow the north–south axis proposed by Yang & Kenagy (2009).

In our geographically expanded sample of mice from Oregon, we again found strong mtDNA divergence (Fig. 3) according to the two major clades (Pacific Northwest and Californian), as previously described (Yang and Kenagy 2009). However, in contrast to the north–south axis of mtDNA differentiation previously described (Yang and Kenagy 2009), mice with Californian mtDNA haplotypes in this geographically expanded sample were less discretely distributed on the geographic landscape (Fig. 4). One formerly admixed location in the contact zone (Malheur County, in southeastern Oregon) was no longer admixed and was composed entirely of Pacific Northwest mtDNA haplotypes. Additionally, one location north of the previously described contact zone (Baker County, in northeastern Oregon) possessed mice with Californian mtDNA haplotypes (Fig. 4). Bootstrap support for the mtDNA split between Pacific Northwest and Californian mice was lower here than previously observed (86% here vs. 95% in Yang and Kenagy [2009]), but the posterior probability of the Pacific Northwest node in the Bayesian analysis was high (0.99). We visually inspected the mtDNA sequence data of each newly sampled mouse for the conserved substitutions associated with the Californian mtDNA haplogroup and found that each mouse with these substitutions was included in the Californian clade of the mtDNA tree. We attribute this difference in bootstrap support to the increased sample size of the present study and the concomitant increase in the amount of variation in sites where these conserved substitutions do not occur.

The locality-based phylogeny inferred from *BEAST did not reveal significant geographic genetic structure among samples from each of the six localities. Nodal posterior probabilities for genetic relationships between localities were quite low (0.1737–0.3482), and the tree as a whole was unresolved (Appendix S3).

### Morphological and nuclear genetic differentiation

**Figure 5:**
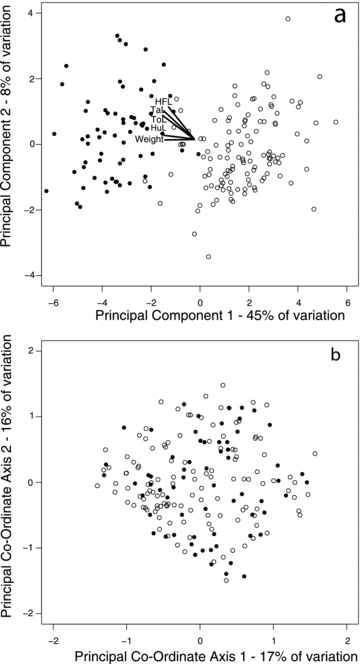
Morphological and genotypic variation in deer mouse populations across the state of Oregon. Individuals from coastal forest habitats are represented by filled circles and individuals from interior sagebrush-steppe habitats by open circles. (A) Principal component analysis of morphological variation. Vectors are shown for the five traits with the highest loadings on PC1: Total length (ToL), Tail length (TaL), Humerus length (HuL), Weight, and Body length (BoL). All vector magnitudes are magnified by 5 for clarity.

Mice from forest and sagebrush-steppe localities were significantly different in overall morphology (Fig. 5A), supporting the hypothesis of an east–west axis of morphological differentiation among Oregon mice. Our PCA revealed that individuals from coastal forest habitats were morphologically distinct overall from sagebrush-steppe individuals. The first principal component axis (PC1) was highly significant both by comparison to the broken-stick distribution (observed variance [eigenvalue] explained = 8.634, expected variance explained = 3.548) and the randomization test (*P* < 0.001). None of the remaining principal component axes were significantly explanatory. Greater PC1 values were correlated with larger measurements of all 19 traits, indicating that PC1 values corresponded to overall size of an individual. However, the range of loadings on PC1 varied greatly across traits (−0.035 to −0.316, mean = −0.219, SD = 0.068), indicating that the size increase in forest populations was allometric rather than isometric in nature and that PC1 values thus reflect variation in body proportions to some degree. The five measurements with the strongest loadings on PC1 were total length (−0.316), humerus length (−0.302), tail length (−0.299), weight (−0.296), and body length (−0.267).

Unlike the PCA for morphological data, the PCoA for microsatellite data found that individuals across the entire state of Oregon (both forest and shrub-steppe habitats) had very similar nuclear microsatellite variation (Fig. 5B). The microsatellite data were highly variable (Appendix S4), and 1.9% of the data were missing. None of the first three principal co-ordinate axes was significantly explanatory under the broken stick distribution (PCoA1: Observed = 17.2%, Expected = 31.4%. PCoA2: Observed = 15.58%, Expected = 20.32%. PCoA3: Observed = 12.17%, Expected = 14.76%). The Mantel test found no correlation between morphological and neutral molecular genetic distance (*r* = −0.03, *P* = 0.827).

### Estimation of migration rates between populations

In both microsatellite and nuclear sequence analyses, all interlocality estimates of θ*m* were significantly different from zero with the exception of migration from Curry into Lincoln and Curry into Wasco Counties in the nuclear sequence analysis (Table 1). Overall, estimates of migration between each locality were quite high in this unrestricted full-migration matrix framework, typically with gene flow equivalent to one or more migrants between each pair of localities per generation. Notably, substantial pairwise gene flow is present between the forest localities (Lincoln, Curry) and the sagebrush-steppe localities (Wasco, Klamath, Baker, Malheur).

**Table 1:** Rates of interpopulation gene flow, given as nonsymmetric interpopulation values of θ*m* (migration) from LAMARC analysis, based on (A) microsatellite data (nine loci) and (B) nuclear sequence data (two loci). Curry and Lincoln Counties are coastal forest localities, whereas Malheur, Wasco, Klamath, and Baker Counties are inland sagebrush-steppe localities. Bold values indicate mean estimates across runs, while values in parentheses indicate mean 95% confidence intervals across runs

(A) Microsatellite data							
To							
		Curry	Lincoln	Malheur	Wasco	Klamath	Baker
From	Curry		**9.03** (3.22–9.59)	**1.19** (1.12–1.26)	**6.37** (6.23–7.07)	**4.13** (3.64–4.41)	**5.5** (4.9–5.5)
	Lincoln	**9.8** (5.4–10.9)		**8.3** (2.3–8.8)	**2.95** (2.77–4.11)	**5.72** (5.45–6.70)	**6.25** (4.91–7.95)
	Malheur	**2.98** (2.14–3.1)	**2.7** (2.61–2.89)		**9.9** (7.74–11.5)	**5.7** (4.66–6.9)	**3.35** (2.8–3.7)
	Wasco	**4.9** (4.31–9.4)	**4.24** (2.53–5.2)	**9.3** (4.24–9.92)		**4.1** (2.8–4.6)	**5.2** (3.90–5.54)
	Klamath	**1.99** (1.9–2.22)	**2.42** (1.8–2.68)	**1.7** (1.27–2.14)	**2.02** (1.55–2.6)		**5.96** (5.36–6.23)
	Baker	**6.49** (2.91–7.06)	**3.99** (3.22–5.71)	**1.71** (1.51–2.02)	**2.34** (1.25–2.85)	**6.23** (60.2–6.64)	

(B) Nuclear sequence data							
To							
		Curry	Lincoln	Malheur	Wasco	Klamath	Baker
From	Curry		**1.16** (<0.01–1.9)	**5.13** (0.80–7.17)	**3.98** (<0.01–4.8)	**1.61** (0.34–2.64)	**3.41** (0.07–5.8)
	Lincoln	**17.7** (0.013–40.8)		**37.4** (2.9–74.8)	**90** (42.8–149)	**127** (3.3–167)	**37.4** (0.04 – 60.8)
	Malheur	**3.02** (0.71 – 6.34)	**1.01** (0.16–2.39)		**1.84** (1.29–3.78)	**1.75** (0.26–3.48)	**2.91** (0.18–4.27)
	Wasco	**109** (0.01–132)	**46** (9.73–87.5)	**52.2** (15.2–97.8)		**20.7** (2.26–45.7)	**3.82** (0.07–41.1)
	Klamath	**67** (17.7–163)	**252** (5.7–362)	**107** (34–267)	**75** (19.8–133)		**134** (0.04–241)
	Baker	**31** (6.87–47.7)	**14.6** (0.02–34.7)	**53** (13.8–96)	**17.9** (0.02–47.7)	**49** (0.05–77.7)	

### Estimation of population number

The STRUCTURE analysis supported the presence of either one or two genetic populations among the six localities sampled. The magnitude of ΔLn P(D) was greatest between *k* = 1 and *k* = 2 and was almost twice as large as the next highest value of ΔLn P(D) (between *k* = 4 and *k* = 5) (Appendix S5). Waterfall diagrams of runs with *k* = 2 did not indicate any population structure, corresponding to neither sampling locality nor mtDNA haplotype, and overall differentiation between these populations was low for these runs (mean genetic distance between populations = 0.03678). The waterfall diagram for the individual run with the highest Ln Likelihood (*k* = 6, run no. 10) also did not indicate any population structure corresponding to sampling locality.

## Discussion

### Mitochondrial DNA and nuclear DNA sequence differentiation

In terms of population delimitation, individuals of both mitochondrial clades were found at the same localities, and thus they have the opportunity to interact demographically. Thus, under the ecological paradigm of Waples and Gaggiotti (2009), mice of both clades form a single population. Based on the nuclear DNA data, mice of both clades are likely interbreeding and thus form a single population under the evolutionary paradigm of Waples and Gaggiotti (2009). We thus conclude that in this case designating mice with distinct mtDNA haplotypes as separate populations is inaccurate.

The mtDNA divergence observed here is consistent with the divergence we originally identified (Yang and Kenagy 2009). However, the nuclear DNA data do not support a monophyletic relationship of individuals with each type of mtDNA, indicating that mtDNA variation is not a reliable indicator of isolation. In fact, overall levels of gene flow between localities across the breadth of Oregon indicate that gene flow is occurring between localities throughout Oregon, and the data from the *BEAST and the PCoA analysis support a single pool of nuclear genetic variation in mice from all these localities. Unfortunately, we cannot use the Δ*K* metric (Evanno et al. 2005) to evaluate the STRUCTURE results under the hypothesis of a single genetic pool (*K* = 1), which was supported by the *BEAST and PCoA analyses. Nevertheless, the two clusters supported by the Δ*K* metric were relatively undifferentiated in terms of *F*_ST_, and this could represent stochastic variation present within a single genetic cluster. Additionally, membership in these two clusters corresponded to neither mtDNA haplogroup nor to locality.

The geographically expanded sampling of the current study reveals that the admixture zone between Pacific Northwest and Californian mtDNA haplotypes may be more geographically complex than the north–south gradient described by Yang and Kenagy (2009). One locality north of the previously identified northern extreme of the mtDNA contact zone possessed Californian haplotypes, while a formerly admixed locality further to the south now possessed exclusively Pacific Northwest haplotypes. This would be contrary to the hypothesis of Yang and Kenagy (2009) that the contact zone has shifted southwards over time following Pleistocene glaciation. However, each of these localities is only represented in the current study by eight individuals, and thus these differences may be stochastic in nature rather than representing a biologically significant shift. Accordingly, these Californian haplotypes may have been present in the previously unsampled Baker county all along and may still be present in Malheur county but were simply not present among the mice sampled from that county in the current study. In any case, our new mtDNA results indicate that the geographic distribution of these haplogroups on the landscape may be quite different from the simple north–south pattern we proposed in 2009.

The locality-based phylogeny of nuclear DNA data was unresolved, indicating that mice from these six localities are not genetically distinct. This finding is concordant with the large amounts of interpopulation gene flow indicated by the LAMARC analysis. As an alternative to using microsatellite data to infer population relationships as in Yang and Kenagy (2009), we had hoped to uncover further detail on population relationships by incorporating models of genetic evolution and inferring coalescent-based branching relationships between individuals in *BEAST and LAMARC, rather than simply clustering individuals using unweighted genetic distances as in the PCoA analysis. Taking these phylogenetic approaches required sequence data in order to generate models of genetic evolution. However, this far more money- and time-intensive method of studying population relationships did not reveal any additional population structure. The overall result of genetic homogeneity, potentially caused by high amounts of gene flow across the breath of Oregon *P. maniculatus* populations, supports the general pattern of genetic homogeneity suggested by Yang and Kenagy (2009) within the mtDNA contact zone.

### Morphological and nuclear genetic differentiation

The morphological and nuclear genetic patterns of differentiation that we observed are discordant (Fig. 5). The morphological pattern falls into two distinct clusters that appear to be strongly associated with the habitat type (forest or shrub-steppe) associated with the samples. We have quantified here this pattern of morphological differentiation in mice across Oregon for the first time with statistical rigor. The original accounts of variation in body size and coloration formed the basis of previous subspecies designations across Oregon (Bailey 1936; Verts and Carraway 1998), which suggested us the possible presence of distinct populations. Here, we must consider the possible causes of this morphological differentiation.

One conclusion would be that these mice simply have a plastic phenotypic response to their local environment and that they could represent a single population. On the other hand, these mice may be undergoing divergent natural selection that results in a pattern of habitat-associated local adaptation. Our results show that forest mice are morphologically differentiated from sagebrush-steppe mice despite substantial gene flow between populations associated with different habitat types, suggesting that gene flow is being balanced by some other evolutionary phenomenon, perhaps divergent natural selection. The pattern of morphological differentiation between Oregon forest and sagebrush-steppe mice is similar to a pattern of morphological differentiation between forest and grassland mice observed in other North American deer mouse populations. In these other populations, mice that live in forested habitats are larger, longer-tailed, and have longer hind feet than mice that live in adjacent nonforested habitat (Clark 1936, Dice 1940, Blair 1950, Horner 1954). These authors postulate that the morphological differentiation of forest mice is an adaptation to an arboreal lifestyle.

In manipulative experiments, Horner (1954) found that greater lengths of tail, body, and hind feet are positively correlated with increased climbing performance. Furthermore, these phenotypic traits associated with climbing have been shown to be highly heritable in the narrow sense (Lofsvold 1986, Thompson 1990). In our study, forest mice had longer bodies (10.09-mm longer, *t* = 12.28, df = 165, *P* < 0.001), longer tails (26.07-mm longer, two-tailed *t*-test, *t* = −25.64, df = 132, *P* <.001), and longer hind feet (2.64-mm longer, two-tailed *t*-test, *t* = −12.70, df = 147, *P* <.001) than interior sagebrush-steppe mice. Coastal forest mice also had longer tails relative to body length than interior mice (17.6% longer relative to body length, two-tailed *t*-test, *t* = −12.89, df = 132, *P* <.001). In contrast to Horner's (1954) study, the nonforest mice sampled here had longer feet relative to body size than forest mice (0.9% longer relative to body size, two-tailed *t*-test, *t* = −3.18, df = 205, *P* = .002). Interestingly, forest and sagebrush-steppe populations had statistically indistinguishable numbers of presacral vertebrae (0.05 mean difference in number of presacral vertebrae, *t* = −0.74, df = 108, *P* = 0.46) despite a large difference in number of tail (postsacral) vertebrae (2.85 more vertebrae in forest mice, two-tailed *t*-test, *t* = −13.31, df = 130, *P* <.001). This increase in body segmentation limited to a specific body structure suggests potential evolution of developmental regulatory genes in forest mice (Economides et al. 2003). A thorough examination of whether morphological differentiation in Oregon *P. maniculatus* results from local adaptation rather than from phenotypic plasticity would require common-garden, reciprocal-transplant, and cross-fostering experiments to determine the genetic, environmental, and maternal-effect components of phenotypic variance in wild populations, which is beyond the scope of the present study. Nevertheless, we would encourage further research in this direction.

### Ecological and evolutionary population paradigms

Our results reflect the inherent disagreement between the ecological and evolutionary population paradigms of Waples and Gagiotti (2005) over the chronological and spatial span of interaction. Ecological population paradigms emphasize co-occurrence in space and time to provide an opportunity for demographic interaction. Evolutionary population paradigms emphasize genetic interactions between individuals that can take place many generations after an individual is deceased through the mating of descendant offspring, allowing genetic continuity over large geographic distances. Delimiting populations under an ecological population paradigm allows a priori population delimitation based solely on geographic location of individuals. In contrast, delimiting populations under an evolutionary paradigm requires (in the absence of direct observation of reproduction) genetic analysis in order to identify genetic relatedness between individuals produced by past reproductive interactions between their ancestors, allowing post facto population delimitation. In the current study, mice from forest and sagebrush-steppe environments form separate ecological populations because of their geographic separation but constitute a single evolutionary population based on their genetic cohesion. This finding highlights the difficulty of using an ecological paradigm to delimit populations of species with geographically broad, continuous distributions such as *P. maniculatus*.

In this study, we were interested primarily in the evolution of the populations we studied, and based on our genetic evidence we conclude that Oregon *P. maniculatus* compose a single population. Specifically, the genes of any individual could eventually mix with the genes of any other Oregon *P. maniculatus* over an evolutionary time frame (perhaps over centuries). However, over an ecological time frame (perhaps over tens of years) this type of reproductive interaction would be unlikely. Thus, if ecological and evolutionary population paradigms are to be reconciled as a single concrete population definition, we will likely have to specify the time scale over which interactions between individuals must occur. For the time being, deciding whether to apply an ecological or evolutionary population paradigm to a research problem should rest with the researcher (Waples and Gaggiotti 2005). Population delimitation based on an evolutionary paradigm allows researchers to integrate genetic evidence of past reproductive interactions between ancestors, resulting in a chronologically expansive body of evidence that may better reflect long-term patterns of population cohesion. Population delimitation based on an ecological paradigm will likely be the more conservative than delimitation based on an evolutionary paradigm simply because of the small spatial and chronological scale over which demographic interactions can take place. More research is necessary on whether genetic methods can reliably reflect demographic interactions (Lowe and Allendorf 2010), and as in the current study the spatial and chronological scale of demographic and reproductive interactions may be incongruent for many populations. Consequently, defining a “population” may remain as difficult as defining a “species” for some time to come.

## Supporting Information

**Appendix S1:** List of individual specimens sampled for this study.

**Appendix S2:** AWTY analysis of MRBAYES analysis convergence.

**Appendix S3:** Locality-based tree of nuclear sequence data.

**Appendix S4:** List of microsatellite loci used in this study.

**Appendix S5:** Results of STRUCTURE runs.
