# Supplementary material for: Population delimitation across contrasting evolutionary clines in deer mice (Peromyscus maniculatus)
Source: Ecol Evol. 2011 Sep;1(1):26–36. doi: 10.1002/ece3.3 (PMC3287378; doi:10.1002/ece3.3)

**Supporting Information**

**Appendix S1**: **List of individual specimens sampled for this study**. University of Washington Burke Museum (UWBM) mammal collection number and preparation number are given for each sample. Asterisks on UWBM numbers indicate individuals included in the nuclear sequence data analysis (eight from each locality, total *n* = 48).

**Appendix S2: AWTY analysis of MRBAYES analysis convergence.**

**Appendix S3**: **Locality-based tree of nuclear sequence data**. Coalescent-based phylogeny of individuals from each locality using data

**Appendix S4: List of microsatellite loci used in this study**. All microsatellite loci deviated from Hardy-Weinberg equilibrium, which we attribute to the large number of alleles at each locus. A Wahlund effect is unlikely given the lack of population structure in genotypic variation.

**Appendix S5:** **Results of STRUCTURE runs.** Summary statistics for STRUCTURE analysis of population number in the microsatellite data. Graphs of k vs. mean Ln P(D) and ΔLn P(D) vs. k comparisons are included as well.

**Appendix S1**: **List of individual specimens sampled for this study.**

| UWBM number | Preparation number | Coll. Day | Coll. Month | Coll. Year | Locality | Lat  (xx.xxxx^o^N) | Long  (xxx.xxxx^o^W) | Habitat |
| --- | --- | --- | --- | --- | --- | --- | --- | --- |
| 79973 | D. S. Yang 210 | 11 | JUL | 2006 | Curry | 423451N | 1242055W | Forest |
| 79974 | D. S. Yang 211 | 11 | JUL | 2006 | Curry | 423451N | 1242055W | Forest |
| 79975 | D. S. Yang 212 | 11 | JUL | 2006 | Curry | 423451N | 1242055W | Forest |
| 79976 | D. S. Yang 213 | 11 | JUL | 2006 | Curry | 423451N | 1242055W | Forest |
| 79977 | D. S. Yang 214 | 11 | JUL | 2006 | Curry | 423451N | 1242055W | Forest |
| 79978 | D. S. Yang 215 | 11 | JUL | 2006 | Curry | 423451N | 1242055W | Forest |
| 79979 | D. S. Yang 216 | 11 | JUL | 2006 | Curry | 423451N | 1242055W | Forest |
| 79980 | D. S. Yang 217 | 11 | JUL | 2006 | Curry | 423451N | 1242055W | Forest |
| 79981 | D. S. Yang 218 | 11 | JUL | 2006 | Curry | 423514N | 1241750W | Forest |
| 79982 | D. S. Yang 219 | 11 | JUL | 2006 | Curry | 423514N | 1241750W | Forest |
| 79983 | D. S. Yang 220 | 11 | JUL | 2006 | Curry | 423514N | 1241750W | Forest |
| 79984 | D. S. Yang 221 | 11 | JUL | 2006 | Curry | 423514N | 1241750W | Forest |
| 79985 | D. S. Yang 222 | 11 | JUL | 2006 | Curry | 423514N | 1241750W | Forest |
| 79986 | D. S. Yang 223 | 11 | JUL | 2006 | Curry | 423514N | 1241750W | Forest |
| 79987 | D. S. Yang 224 | 11 | JUL | 2006 | Curry | 423514N | 1241750W | Forest |
| 79988 | D. S. Yang 225 | 11 | JUL | 2006 | Curry | 423514N | 1241750W | Forest |
| 80044 | D. S. Yang 308 | 23 | SEP | 2006 | Malheur | 421838N | 1175021W | Sagebrush |
| 80045 | D. S. Yang 309 | 23 | SEP | 2006 | Malheur | 421838N | 1175021W | Sagebrush |
| 80046 | D. S. Yang 310 | 23 | SEP | 2006 | Malheur | 421838N | 1175021W | Sagebrush |
| 80047 | D. S. Yang 311 | 23 | SEP | 2006 | Malheur | 421838N | 1175021W | Sagebrush |
| 80048 | D. S. Yang 312 | 23 | SEP | 2006 | Malheur | 421838N | 1175021W | Sagebrush |
| 80049 | D. S. Yang 313 | 23 | SEP | 2006 | Malheur | 421838N | 1175021W | Sagebrush |
| 80050 | D. S. Yang 314 | 23 | SEP | 2006 | Malheur | 421838N | 1175021W | Sagebrush |
| 80051 | D. S. Yang 315 | 23 | SEP | 2006 | Malheur | 421838N | 1175021W | Sagebrush |
| 80052 | D. S. Yang 316 | 23 | SEP | 2006 | Malheur | 421838N | 1175021W | Sagebrush |
| 80053 | D. S. Yang 317 | 23 | SEP | 2006 | Malheur | 421838N | 1175021W | Sagebrush |
| 80054 | D. S. Yang 318 | 23 | SEP | 2006 | Malheur | 421838N | 1175021W | Sagebrush |
| 80055 | D. S. Yang 319 | 23 | SEP | 2006 | Malheur | 421838N | 1175021W | Sagebrush |
| 80056 | D. S. Yang 320 | 23 | SEP | 2006 | Malheur | 421838N | 1175021W | Sagebrush |
| 80057 | D. S. Yang 321 | 23 | SEP | 2006 | Malheur | 421838N | 1175021W | Sagebrush |
| 80058 | D. S. Yang 322 | 23 | SEP | 2006 | Malheur | 421838N | 1175021W | Sagebrush |
| 80059 | D. S. Yang 323 | 23 | SEP | 2006 | Malheur | 421838N | 1175021W | Sagebrush |
| 80060 | D. S. Yang 324 | 23 | SEP | 2006 | Malheur | 421838N | 1175021W | Sagebrush |
| 80061 | D. S. Yang 325 | 23 | SEP | 2006 | Malheur | 421838N | 1175021W | Sagebrush |
| 80062 | D. S. Yang 326 | 23 | SEP | 2006 | Malheur | 421838N | 1175021W | Sagebrush |
| 80063 | D. S. Yang 332 | 23 | SEP | 2006 | Malheur | 421838N | 1175021W | Sagebrush |
| 80099 | D. S. Yang 372 | 23 | SEP | 2006 | Malheur | 421838N | 1175021W | Sagebrush |
| 80100* | D. S. Yang 374* | 20 | JUN | 2007 | Lincoln | 445433N | 1235479W | Forest |
| 80101* | D. S. Yang 375* | 20 | JUN | 2007 | Lincoln | 445433N | 1235479W | Forest |
| 80102* | D. S. Yang 376* | 20 | JUN | 2007 | Lincoln | 445433N | 1235479W | Forest |
| 80103* | D. S. Yang 377* | 20 | JUN | 2007 | Lincoln | 445433N | 1235479W | Forest |
| 80104* | D. S. Yang 378* | 20 | JUN | 2007 | Lincoln | 445433N | 1235479W | Forest |
| 80105* | D. S. Yang 379* | 21 | JUN | 2007 | Lincoln | 445577N | 1235604W | Forest |
| 80106* | D. S. Yang 380* | 21 | JUN | 2007 | Lincoln | 445577N | 1235604W | Forest |
| 80107* | D. S. Yang 381* | 21 | JUN | 2007 | Lincoln | 445577N | 1235604W | Forest |
| 80108 | D. S. Yang 382 | 21 | JUN | 2007 | Lincoln | 445577N | 1235604W | Forest |
| 80109 | D. S. Yang 383 | 21 | JUN | 2007 | Lincoln | 445577N | 1235604W | Forest |
| 80110 | D. S. Yang 385 | 22 | JUN | 2007 | Lincoln | 445577N | 1235604W | Forest |
| 80111 | D. S. Yang 390 | 23 | JUN | 2007 | Lincoln | 450252N | 1235540W | Forest |
| 80112 | D. S. Yang 391 | 23 | JUN | 2007 | Lincoln | 450252N | 1235540W | Forest |
| 80113 | D. S. Yang 392 | 23 | JUN | 2007 | Lincoln | 450252N | 1235540W | Forest |
| 80114 | D. S. Yang 393 | 23 | JUN | 2007 | Lincoln | 450252N | 1235540W | Forest |
| 80115 | D. S. Yang 394 | 23 | JUN | 2007 | Lincoln | 450252N | 1235540W | Forest |
| 80116 | D. S. Yang 395 | 23 | JUN | 2007 | Lincoln | 450252N | 1235540W | Forest |
| 80117 | D. S. Yang 396 | 23 | JUN | 2007 | Lincoln | 450252N | 1235540W | Forest |
| 80118 | D. S. Yang 397 | 23 | JUN | 2007 | Lincoln | 450252N | 1235540W | Forest |
| 80119 | D. S. Yang 398 | 23 | JUN | 2007 | Lincoln | 450252N | 1235540W | Forest |
| 80120 | D. S. Yang 401 | 23 | JUN | 2007 | Lincoln | 450265N | 1235471W | Forest |
| 80121 | D. S. Yang 402 | 23 | JUN | 2007 | Lincoln | 450265N | 1235471W | Forest |
| 80122 | D. S. Yang 403 | 23 | JUN | 2007 | Lincoln | 450265N | 1235471W | Forest |
| 80123 | D. S. Yang 404 | 23 | JUN | 2007 | Lincoln | 450265N | 1235471W | Forest |
| 80124 | D. S. Yang 405 | 23 | JUN | 2007 | Lincoln | 450265N | 1235471W | Forest |
| 80125 | D. S. Yang 406 | 23 | JUN | 2007 | Lincoln | 450265N | 1235471W | Forest |
| 80126 | D. S. Yang 407 | 23 | JUN | 2007 | Lincoln | 450265N | 1235471W | Forest |
| 80127 | D. S. Yang 408 | 23 | JUN | 2007 | Lincoln | 450265N | 1235471W | Forest |
| 80128* | D. S. Yang 412* | 24 | JUN | 2007 | Curry | 423451N | 1242056W | Forest |
| 80129* | D. S. Yang 413* | 24 | JUN | 2007 | Curry | 423451N | 1242056W | Forest |
| 80130* | D. S. Yang 414* | 24 | JUN | 2007 | Curry | 423451N | 1242056W | Forest |
| 80131* | D. S. Yang 415* | 24 | JUN | 2007 | Curry | 423451N | 1242056W | Forest |
| 80132* | D. S. Yang 416* | 24 | JUN | 2007 | Curry | 423451N | 1242056W | Forest |
| 80133* | D. S. Yang 417* | 24 | JUN | 2007 | Curry | 423451N | 1242056W | Forest |
| 80134* | D. S. Yang 418* | 24 | JUN | 2007 | Curry | 423451N | 1242056W | Forest |
| 80135* | D. S. Yang 419* | 24 | JUN | 2007 | Curry | 423451N | 1242056W | Forest |
| 80136 | D. S. Yang 420 | 24 | JUN | 2007 | Curry | 423451N | 1242056W | Forest |
| 80137 | D. S. Yang 421 | 24 | JUN | 2007 | Curry | 423451N | 1242056W | Forest |
| 80138 | D. S. Yang 422 | 24 | JUN | 2007 | Curry | 423451N | 1242056W | Forest |
| 80139 | D. S. Yang 423 | 24 | JUN | 2007 | Curry | 423451N | 1242056W | Forest |
| 80140 | D. S. Yang 424 | 24 | JUN | 2007 | Curry | 423451N | 1242056W | Forest |
| 80141 | D. S. Yang 425 | 24 | JUN | 2007 | Curry | 423451N | 1242056W | Forest |
| 80142 | D. S. Yang 426 | 24 | JUN | 2007 | Curry | 423451N | 1242056W | Forest |
| 80143 | D. S. Yang 427 | 24 | JUN | 2007 | Curry | 423451N | 1242056W | Forest |
| 80144 | D. S. Yang 428 | 24 | JUN | 2007 | Curry | 423451N | 1242056W | Forest |
| 80145 | D. S. Yang 429 | 24 | JUN | 2007 | Curry | 423451N | 1242056W | Forest |
| 80146 | D. S. Yang 430 | 25 | JUN | 2007 | Curry | 423451N | 1242056W | Forest |
| 80147 | D. S. Yang 431 | 25 | JUN | 2007 | Curry | 423451N | 1242056W | Forest |
| 80148 | D. S. Yang 432 | 25 | JUN | 2007 | Curry | 423451N | 1242056W | Forest |
| 80149 | D. S. Yang 433 | 25 | JUN | 2007 | Curry | 423451N | 1242056W | Forest |
| 80150 | D. S. Yang 434 | 25 | JUN | 2007 | Curry | 423451N | 1242056W | Forest |
| 80151 | D. S. Yang 435 | 25 | JUN | 2007 | Curry | 423451N | 1242056W | Forest |
| 80152 | D. S. Yang 436 | 25 | JUN | 2007 | Curry | 423451N | 1242056W | Forest |
| 80153 | D. S. Yang 437 | 25 | JUN | 2007 | Curry | 423451N | 1242056W | Forest |
| 80154 | D. S. Yang 438 | 25 | JUN | 2007 | Curry | 423451N | 1242056W | Forest |
| 80155 | D. S. Yang 439 | 25 | JUN | 2007 | Curry | 423451N | 1242056W | Forest |
| 80156 | D. S. Yang 440 | 25 | JUN | 2007 | Curry | 423451N | 1242056W | Forest |
| 80157 | D. S. Yang 441 | 25 | JUN | 2007 | Curry | 423451N | 1242056W | Forest |
| 80158 | D. S. Yang 442 | 25 | JUN | 2007 | Curry | 423451N | 1242056W | Forest |
| 80159 | D. S. Yang 444 | 26 | JUN | 2007 | Lincoln | 450252N | 1235540W | Forest |
| 80160 | D. S. Yang 445 | 26 | JUN | 2007 | Lincoln | 450252N | 1235540W | Forest |
| 80161 | D. S. Yang 446 | 26 | JUN | 2007 | Lincoln | 450252N | 1235540W | Forest |
| 80162 | D. S. Yang 447 | 26 | JUN | 2007 | Lincoln | 450252N | 1235540W | Forest |
| 80163 | D. S. Yang 448 | 26 | JUN | 2007 | Lincoln | 450252N | 1235540W | Forest |
| 80164 | D. S. Yang 449 | 26 | JUN | 2007 | Lincoln | 450252N | 1235540W | Forest |
| 80165 | D. S. Yang 450 | 26 | JUN | 2007 | Lincoln | 450252N | 1235540W | Forest |
| 80166 | D. S. Yang 451 | 26 | JUN | 2007 | Lincoln | 450252N | 1235540W | Forest |
| 80167 | D. S. Yang 452 | 26 | JUN | 2007 | Lincoln | 450252N | 1235540W | Forest |
| 80168 | D. S. Yang 453 | 26 | JUN | 2007 | Lincoln | 450252N | 1235540W | Forest |
| 80169 | D. S. Yang 454 | 26 | JUN | 2007 | Lincoln | 450252N | 1235540W | Forest |
| 80170 | D. S. Yang 455 | 26 | JUN | 2007 | Lincoln | 450252N | 1235540W | Forest |
| 80171 | D. S. Yang 456 | 26 | JUN | 2007 | Lincoln | 450252N | 1235540W | Forest |
| 80172 | D. S. Yang 460 | 3 | JUL | 2007 | Wasco | 445401N | 1202682W | Sagebrush |
| 80173* | D. S. Yang 461* | 3 | JUL | 2007 | Wasco | 445401N | 1202682W | Sagebrush |
| 80174* | D. S. Yang 462* | 3 | JUL | 2007 | Wasco | 445401N | 1202682W | Sagebrush |
| 80175* | D. S. Yang 463* | 3 | JUL | 2007 | Wasco | 445401N | 1202682W | Sagebrush |
| 80176* | D. S. Yang 464* | 3 | JUL | 2007 | Wasco | 445401N | 1202682W | Sagebrush |
| 80177* | D. S. Yang 465* | 3 | JUL | 2007 | Wasco | 445401N | 1202682W | Sagebrush |
| 80178* | D. S. Yang 466* | 3 | JUL | 2007 | Wasco | 445401N | 1202682W | Sagebrush |
| 80179* | D. S. Yang 467* | 12 | JUL | 2007 | Wasco | 444669N | 1205670W | Sagebrush |
| 80180* | D. S. Yang 468* | 12 | JUL | 2007 | Wasco | 444669N | 1205670W | Sagebrush |
| 80181 | D. S. Yang 469 | 12 | JUL | 2007 | Wasco | 444669N | 1205670W | Sagebrush |
| 80182 | D. S. Yang 470 | 12 | JUL | 2007 | Wasco | 444669N | 1205670W | Sagebrush |
| 80183 | D. S. Yang 471 | 12 | JUL | 2007 | Wasco | 444669N | 1205670W | Sagebrush |
| 80184 | D. S. Yang 472 | 12 | JUL | 2007 | Wasco | 444669N | 1205670W | Sagebrush |
| 80185 | D. S. Yang 473 | 12 | JUL | 2007 | Wasco | 444669N | 1205670W | Sagebrush |
| 80186 | D. S. Yang 474 | 12 | JUL | 2007 | Wasco | 444669N | 1205670W | Sagebrush |
| 80187 | D. S. Yang 475 | 12 | JUL | 2007 | Wasco | 444669N | 1205670W | Sagebrush |
| 80188 | D. S. Yang 476 | 12 | JUL | 2007 | Wasco | 444669N | 1205670W | Sagebrush |
| 80189 | D. S. Yang 477 | 12 | JUL | 2007 | Wasco | 444669N | 1205670W | Sagebrush |
| 80190 | D. S. Yang 478 | 12 | JUL | 2007 | Wasco | 444669N | 1205670W | Sagebrush |
| 80191 | D. S. Yang 479 | 12 | JUL | 2007 | Wasco | 444669N | 1205670W | Sagebrush |
| 80192 | D. S. Yang 480 | 13 | JUL | 2007 | Wasco | 445401N | 1202682W | Sagebrush |
| 80193 | D. S. Yang 481 | 5 | JUL | 2007 | Klamath | 422818N | 1210146W | Sagebrush |
| 80194 | D. S. Yang 482 | 5 | JUL | 2007 | Klamath | 422818N | 1210146W | Sagebrush |
| 80195* | D. S. Yang 483* | 5 | JUL | 2007 | Klamath | 422818N | 1210146W | Sagebrush |
| 80196* | D. S. Yang 484* | 5 | JUL | 2007 | Klamath | 422818N | 1210146W | Sagebrush |
| 80197* | D. S. Yang 485* | 5 | JUL | 2007 | Klamath | 422818N | 1210146W | Sagebrush |
| 80198* | D. S. Yang 486* | 5 | JUL | 2007 | Klamath | 422818N | 1210146W | Sagebrush |
| 80199* | D. S. Yang 487* | 5 | JUL | 2007 | Klamath | 422818N | 1210146W | Sagebrush |
| 80200 | D. S. Yang 488 | 5 | JUL | 2007 | Klamath | 422818N | 1210146W | Sagebrush |
| 80201* | D. S. Yang 489* | 5 | JUL | 2007 | Klamath | 422818N | 1210146W | Sagebrush |
| 80202 | D. S. Yang 490 | 5 | JUL | 2007 | Klamath | 422818N | 1210146W | Sagebrush |
| 80203* | D. S. Yang 491* | 5 | JUL | 2007 | Klamath | 422818N | 1210146W | Sagebrush |
| 80204* | D. S. Yang 492* | 5 | JUL | 2007 | Klamath | 422818N | 1210146W | Sagebrush |
| 80205 | D. S. Yang 493 | 5 | JUL | 2007 | Klamath | 422818N | 1210146W | Sagebrush |
| 80206 | D. S. Yang 494 | 5 | JUL | 2007 | Klamath | 422818N | 1210146W | Sagebrush |
| 80207 | D. S. Yang 495 | 5 | JUL | 2007 | Klamath | 422818N | 1210146W | Sagebrush |
| 80208 | D. S. Yang 496 | 5 | JUL | 2007 | Klamath | 422818N | 1210146W | Sagebrush |
| 80209 | D. S. Yang 497 | 5 | JUL | 2007 | Klamath | 422818N | 1210146W | Sagebrush |
| 80210 | D. S. Yang 498 | 5 | JUL | 2007 | Klamath | 422818N | 1210146W | Sagebrush |
| 80211 | D. S. Yang 499 | 5 | JUL | 2007 | Klamath | 422818N | 1210146W | Sagebrush |
| 80212 | D. S. Yang 500 | 5 | JUL | 2007 | Klamath | 422818N | 1210146W | Sagebrush |
| 80213 | D. S. Yang 501 | 5 | JUL | 2007 | Klamath | 422818N | 1210146W | Sagebrush |
| 80214 | D. S. Yang 502 | 5 | JUL | 2007 | Klamath | 422818N | 1210146W | Sagebrush |
| 80215 | D. S. Yang 503 | 5 | JUL | 2007 | Klamath | 422818N | 1210146W | Sagebrush |
| 80216 | D. S. Yang 504 | 5 | JUL | 2007 | Klamath | 422818N | 1210146W | Sagebrush |
| 80217 | D. S. Yang 505 | 5 | JUL | 2007 | Klamath | 422818N | 1210146W | Sagebrush |
| 80218 | D. S. Yang 506 | 5 | JUL | 2007 | Klamath | 422818N | 1210146W | Sagebrush |
| 80219 | D. S. Yang 507 | 5 | JUL | 2007 | Klamath | 422818N | 1210146W | Sagebrush |
| 80220 | D. S. Yang 508 | 5 | JUL | 2007 | Klamath | 422818N | 1210146W | Sagebrush |
| 80221 | D. S. Yang 509 | 5 | JUL | 2007 | Klamath | 422818N | 1210146W | Sagebrush |
| 80222 | D. S. Yang 510 | 5 | JUL | 2007 | Klamath | 422818N | 1210146W | Sagebrush |
| 80223 | D. S. Yang 511 | 5 | JUL | 2007 | Klamath | 422818N | 1210146W | Sagebrush |
| 80224 | D. S. Yang 512 | 5 | JUL | 2007 | Klamath | 422818N | 1210146W | Sagebrush |
| 80225 | D. S. Yang 513 | 5 | JUL | 2007 | Klamath | 422818N | 1210146W | Sagebrush |
| 80226 | D. S. Yang 514 | 5 | JUL | 2007 | Klamath | 422818N | 1210146W | Sagebrush |
| 80227 | D. S. Yang 515 | 5 | JUL | 2007 | Klamath | 422818N | 1210146W | Sagebrush |
| 80228 | D. S. Yang 516 | 5 | JUL | 2007 | Klamath | 422818N | 1210146W | Sagebrush |
| 80229 | D. S. Yang 517 | 5 | JUL | 2007 | Klamath | 422818N | 1210146W | Sagebrush |
| 80230 | D. S. Yang 518 | 5 | JUL | 2007 | Klamath | 422818N | 1210146W | Sagebrush |
| 80231 | D. S. Yang 519 | 5 | JUL | 2007 | Klamath | 422818N | 1210146W | Sagebrush |
| 80232 | D. S. Yang 520 | 5 | JUL | 2007 | Klamath | 422818N | 1210146W | Sagebrush |
| 80233 | D. S. Yang 521 | 5 | JUL | 2007 | Klamath | 422818N | 1210146W | Sagebrush |
| 80234 | D. S. Yang 522 | 5 | JUL | 2007 | Klamath | 422818N | 1210146W | Sagebrush |
| 80235 | D. S. Yang 523 | 5 | JUL | 2007 | Klamath | 422818N | 1210146W | Sagebrush |
| 80236 | D. S. Yang 524 | 5 | JUL | 2007 | Klamath | 422818N | 1210146W | Sagebrush |
| 80237 | D. S. Yang 525 | 5 | JUL | 2007 | Klamath | 422818N | 1210146W | Sagebrush |
| 80238 | D. S. Yang 526 | 5 | JUL | 2007 | Klamath | 422818N | 1210146W | Sagebrush |
| 80239 | D. S. Yang 527 | 5 | JUL | 2007 | Klamath | 422818N | 1210146W | Sagebrush |
| 80240 | D. S. Yang 528 | 5 | JUL | 2007 | Klamath | 422818N | 1210146W | Sagebrush |
| 80241 | D. S. Yang 529 | 5 | JUL | 2007 | Klamath | 422818N | 1210146W | Sagebrush |
| 80242* | D. S. Yang 531* | 6 | JUL | 2007 | Malheur | 421838N | 1175021W | Sagebrush |
| 80243 | D. S. Yang 532 | 6 | JUL | 2007 | Malheur | 421838N | 1175021W | Sagebrush |
| 80244 | D. S. Yang 533 | 6 | JUL | 2007 | Malheur | 421838N | 1175021W | Sagebrush |
| 80245 | D. S. Yang 534 | 6 | JUL | 2007 | Malheur | 421838N | 1175021W | Sagebrush |
| 80246 | D. S. Yang 535 | 6 | JUL | 2007 | Malheur | 421838N | 1175021W | Sagebrush |
| 80247 | D. S. Yang 536 | 6 | JUL | 2007 | Malheur | 421838N | 1175021W | Sagebrush |
| 80248 | D. S. Yang 537 | 6 | JUL | 2007 | Malheur | 421838N | 1175021W | Sagebrush |
| 80249 | D. S. Yang 538 | 6 | JUL | 2007 | Malheur | 421838N | 1175021W | Sagebrush |
| 80250 | D. S. Yang 539 | 6 | JUL | 2007 | Malheur | 421838N | 1175021W | Sagebrush |
| 80251* | D. S. Yang 540* | 7 | JUL | 2007 | Malheur | 421838N | 1175021W | Sagebrush |
| 80252* | D. S. Yang 541* | 7 | JUL | 2007 | Malheur | 421838N | 1175021W | Sagebrush |
| 80253* | D. S. Yang 542* | 7 | JUL | 2007 | Malheur | 421838N | 1175021W | Sagebrush |
| 80254* | D. S. Yang 543* | 7 | JUL | 2007 | Malheur | 421838N | 1175021W | Sagebrush |
| 80255 | D. S. Yang 544 | 7 | JUL | 2007 | Malheur | 421838N | 1175021W | Sagebrush |
| 80256 | D. S. Yang 545 | 7 | JUL | 2007 | Malheur | 421838N | 1175021W | Sagebrush |
| 80257* | D. S. Yang 546* | 7 | JUL | 2007 | Malheur | 421838N | 1175021W | Sagebrush |
| 80258 | D. S. Yang 547 | 7 | JUL | 2007 | Malheur | 421838N | 1175021W | Sagebrush |
| 80259* | D. S. Yang 548* | 7 | JUL | 2007 | Malheur | 421838N | 1175021W | Sagebrush |
| 80260* | D. S. Yang 549* | 7 | JUL | 2007 | Malheur | 421838N | 1175021W | Sagebrush |
| 80261 | D. S. Yang 550 | 7 | JUL | 2007 | Malheur | 421838N | 1175021W | Sagebrush |
| 80262 | D. S. Yang 551 | 7 | JUL | 2007 | Malheur | 421838N | 1175021W | Sagebrush |
| 80263 | D. S. Yang 552 | 7 | JUL | 2007 | Malheur | 421838N | 1175021W | Sagebrush |
| 80264 | D. S. Yang 553 | 7 | JUL | 2007 | Malheur | 421838N | 1175021W | Sagebrush |
| 80265* | D. S. Yang 554* | 8 | JUL | 2007 | Baker | 444845N | 1174059W | Sagebrush |
| 80266* | D. S. Yang 555* | 8 | JUL | 2007 | Baker | 444845N | 1174059W | Sagebrush |
| 80267* | D. S. Yang 556* | 8 | JUL | 2007 | Baker | 444845N | 1174059W | Sagebrush |
| 80268* | D. S. Yang 557* | 8 | JUL | 2007 | Baker | 444845N | 1174059W | Sagebrush |
| 80269* | D. S. Yang 558* | 8 | JUL | 2007 | Baker | 444845N | 1174059W | Sagebrush |
| 80270* | D. S. Yang 559* | 8 | JUL | 2007 | Baker | 444845N | 1174059W | Sagebrush |
| 80271* | D. S. Yang 560* | 8 | JUL | 2007 | Baker | 444845N | 1174059W | Sagebrush |
| 80272* | D. S. Yang 561* | 8 | JUL | 2007 | Baker | 444845N | 1174059W | Sagebrush |
| 80273 | D. S. Yang 562 | 8 | JUL | 2007 | Baker | 444845N | 1174059W | Sagebrush |
| 80274 | D. S. Yang 563 | 8 | JUL | 2007 | Baker | 444845N | 1174059W | Sagebrush |
| 80275 | D. S. Yang 564 | 8 | JUL | 2007 | Baker | 444845N | 1174059W | Sagebrush |
| 80276 | D. S. Yang 565 | 8 | JUL | 2007 | Baker | 444845N | 1174059W | Sagebrush |
| 80277 | D. S. Yang 566 | 8 | JUL | 2007 | Baker | 444845N | 1174059W | Sagebrush |
| 80278 | D. S. Yang 567 | 8 | JUL | 2007 | Baker | 444845N | 1174059W | Sagebrush |
| 80279 | D. S. Yang 568 | 8 | JUL | 2007 | Baker | 444845N | 1174059W | Sagebrush |
| 80280 | D. S. Yang 569 | 8 | JUL | 2007 | Baker | 444845N | 1174059W | Sagebrush |
| 80281 | D. S. Yang 570 | 8 | JUL | 2007 | Baker | 444845N | 1174059W | Sagebrush |
| 80282 | D. S. Yang 571 | 8 | JUL | 2007 | Baker | 444845N | 1174059W | Sagebrush |
| 80283 | D. S. Yang 572 | 8 | JUL | 2007 | Baker | 444845N | 1174059W | Sagebrush |
| 80284 | D. S. Yang 573 | 8 | JUL | 2007 | Baker | 444845N | 1174059W | Sagebrush |
| 80285 | D. S. Yang 574 | 8 | JUL | 2007 | Baker | 444845N | 1174059W | Sagebrush |
| 80286 | D. S. Yang 575 | 8 | JUL | 2007 | Baker | 444845N | 1174059W | Sagebrush |
| 80287 | D. S. Yang 576 | 8 | JUL | 2007 | Baker | 444845N | 1174059W | Sagebrush |
| 80288 | D. S. Yang 577 | 8 | JUL | 2007 | Baker | 444845N | 1174059W | Sagebrush |
| 80289 | D. S. Yang 578 | 8 | JUL | 2007 | Baker | 444845N | 1174059W | Sagebrush |
| 80290 | D. S. Yang 579 | 8 | JUL | 2007 | Baker | 444845N | 1174059W | Sagebrush |
| 80291 | D. S. Yang 580 | 9 | JUL | 2007 | Baker | 444845N | 1174059W | Sagebrush |
| 80292 | D. S. Yang 581 | 9 | JUL | 2007 | Baker | 444845N | 1174059W | Sagebrush |
| 80293 | D. S. Yang 582 | 9 | JUL | 2007 | Baker | 444845N | 1174059W | Sagebrush |
| 80294 | D. S. Yang 583 | 9 | JUL | 2007 | Baker | 444845N | 1174059W | Sagebrush |
| 80295 | D. S. Yang 584 | 9 | JUL | 2007 | Baker | 444845N | 1174059W | Sagebrush |
| 80296 | D. S. Yang 585 | 9 | JUL | 2007 | Baker | 444845N | 1174059W | Sagebrush |
| 80297 | D. S. Yang 586 | 9 | JUL | 2007 | Baker | 444845N | 1174059W | Sagebrush |
| 80298 | D. S. Yang 587 | 6 | JUL | 2007 | Baker | 444845N | 1174059W | Sagebrush |
| 80299 | D. S. Yang 588 | 9 | JUL | 2007 | Baker | 444845N | 1174059W | Sagebrush |
| 80300 | D. S. Yang 589 | 9 | JUL | 2007 | Baker | 444845N | 1174059W | Sagebrush |
| 80301 | D. S. Yang 590 | 9 | JUL | 2007 | Baker | 444845N | 1174059W | Sagebrush |
| 80302 | D. S. Yang 591 | 9 | JUL | 2007 | Baker | 444845N | 1174059W | Sagebrush |
| 80303 | D. S. Yang 592 | 9 | JUL | 2007 | Baker | 444845N | 1174059W | Sagebrush |
| 80304 | D. S. Yang 593 | 9 | JUL | 2007 | Baker | 444845N | 1174059W | Sagebrush |
| 80305 | D. S. Yang 594 | 9 | JUL | 2007 | Baker | 444845N | 1174059W | Sagebrush |
| 80306 | D. S. Yang 595 | 9 | JUL | 2007 | Baker | 444845N | 1174059W | Sagebrush |
| 80307 | D. S. Yang 596 | 9 | JUL | 2007 | Baker | 444845N | 1174059W | Sagebrush |
| 80308 | D. S. Yang 597 | 9 | JUL | 2007 | Baker | 444845N | 1174059W | Sagebrush |
| 80309 | D. S. Yang 598 | 9 | JUL | 2007 | Baker | 444845N | 1174059W | Sagebrush |
| 80310 | D. S. Yang 599 | 9 | JUL | 2007 | Baker | 444845N | 1174059W | Sagebrush |
| 80311 | D. S. Yang 600 | 9 | JUL | 2007 | Baker | 444845N | 1174059W | Sagebrush |
| 80312 | D. S. Yang 601 | 9 | JUL | 2007 | Baker | 444845N | 1174059W | Sagebrush |
| 80313 | D. S. Yang 602 | 9 | JUL | 2007 | Baker | 444845N | 1174059W | Sagebrush |
| 80314 | D. S. Yang 604 | 13 | JUL | 2007 | Wasco | 445401N | 1202682W | Sagebrush |
| 80315 | D. S. Yang 605 | 13 | JUL | 2007 | Wasco | 445401N | 1202682W | Sagebrush |
| 80316 | D. S. Yang 606 | 13 | JUL | 2007 | Wasco | 445401N | 1202682W | Sagebrush |
| 80317 | D. S. Yang 607 | 13 | JUL | 2007 | Wasco | 445401N | 1202682W | Sagebrush |
| 80318 | D. S. Yang 608 | 13 | JUL | 2007 | Wasco | 445401N | 1202682W | Sagebrush |
| 80319 | D. S. Yang 609 | 13 | JUL | 2007 | Wasco | 445401N | 1202682W | Sagebrush |
| 80320 | D. S. Yang 610 | 13 | JUL | 2007 | Wasco | 445401N | 1202682W | Sagebrush |
| 80321 | D. S. Yang 611 | 13 | JUL | 2007 | Wasco | 445401N | 1202682W | Sagebrush |
| 80322 | D. S. Yang 612 | 13 | JUL | 2007 | Wasco | 445401N | 1202682W | Sagebrush |
| 80323 | D. S. Yang 613 | 13 | JUL | 2007 | Wasco | 445401N | 1202682W | Sagebrush |
| 80324 | D. S. Yang 614 | 14 | JUL | 2007 | Wasco | 445127N | 1205193W | Sagebrush |
| 80325 | D. S. Yang 615 | 14 | JUL | 2007 | Wasco | 445227N | 1205193W | Sagebrush |
| 80326 | D. S. Yang 616 | 14 | JUL | 2007 | Wasco | 445227N | 1205193W | Sagebrush |
| 80327 | D. S. Yang 617 | 14 | JUL | 2007 | Wasco | 445227N | 1205193W | Sagebrush |
| 80328 | D. S. Yang 618 | 14 | JUL | 2007 | Wasco | 445227N | 1205193W | Sagebrush |
| 80329 | D. S. Yang 619 | 14 | JUL | 2007 | Wasco | 445227N | 1205193W | Sagebrush |
| 80330 | D. S. Yang 620 | 14 | JUL | 2007 | Wasco | 445227N | 1205193W | Sagebrush |
| 80331 | D. S. Yang 621 | 14 | JUL | 2007 | Wasco | 445227N | 1205193W | Sagebrush |
| 80332 | D. S. Yang 622 | 14 | JUL | 2007 | Wasco | 445227N | 1205193W | Sagebrush |
| 80333 | D. S. Yang 623 | 23 | JUN | 2007 | Curry | 423451N | 1242056W | Forest |

**Appendix S2: AWTY analysis of MRBAYES analysis convergence.**


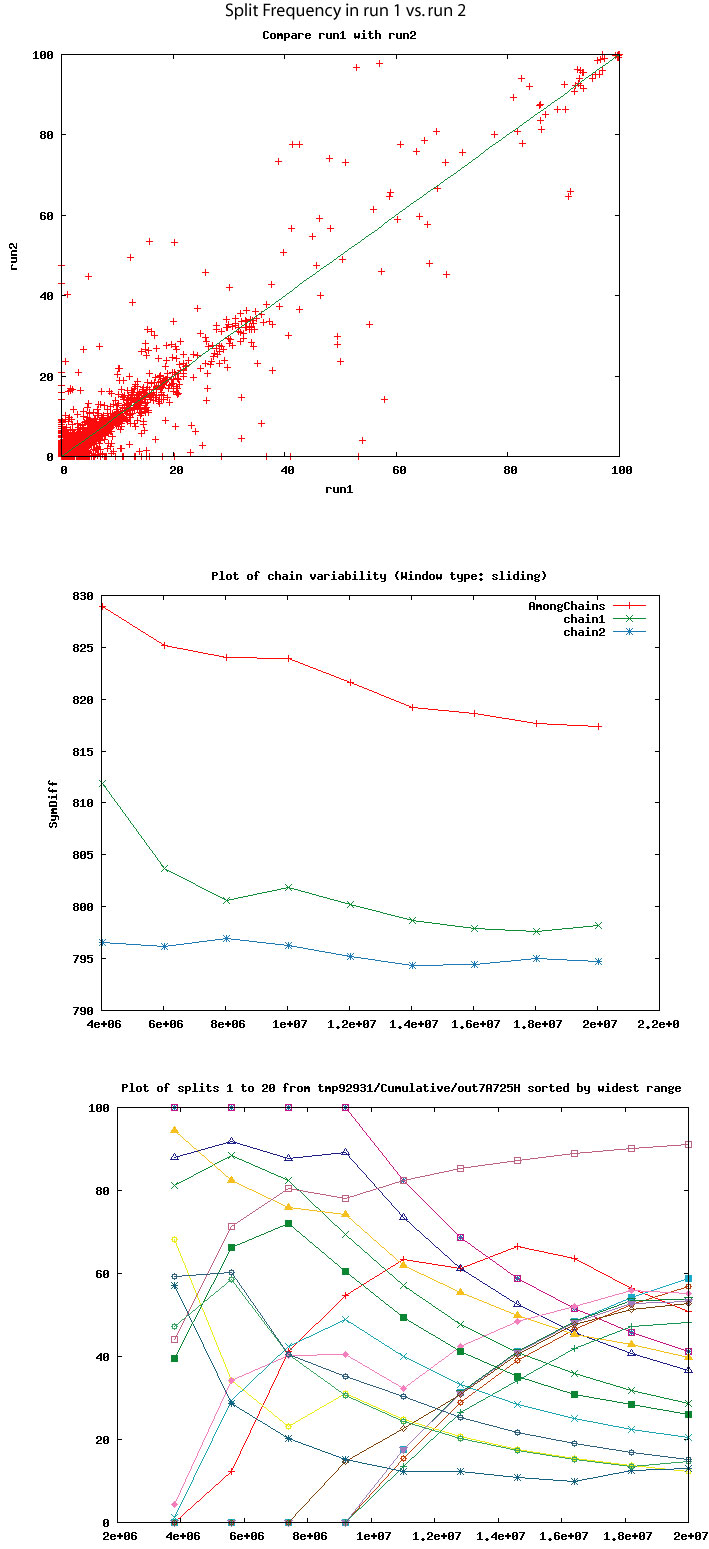


**Appendix S3: Locality-based nuclear gene phylogeny**

**
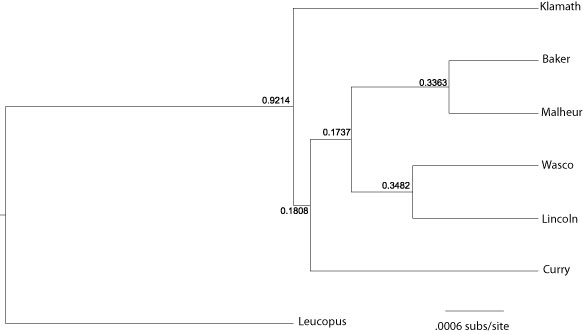
**

**Appendix S4: List of microsatellite loci used in this study**.

| Locus | GenBank Accession | Observed Heterozygosity | Expected Heterozygosity | Number of alleles | Allele Size Range |
| --- | --- | --- | --- | --- | --- |
| BW2-110 | AF526099 | 0.717 | 0.933 | 24 | 90-181 |
| PO-105 | AF380242 | 0.521 | 0.954 | 31 | 115-202 |
| PO-35 | AF380238 | 0.523 | 0.851 | 27 | 239-347 |
| PO-85 | AF380250 | 0.777 | 0.899 | 21 | 190-265 |
| PO-9 | AF380232 | 0.685 | 0.908 | 24 | 137-207 |
| PO3-25 | AY053425 | 0.684 | 0.965 | 35 | 190-279 |
| PO3-68 | AF380248 | 0.851 | 0.948 | 28 | 225-301 |
| PO-21 | AF380233 | 0.599 | 0.831 | 25 | 106-233 |
| PO-40 | AF380239 | 0.786 | 0.972 | 40 | 207-342 |

**Appendix S5:** **Results of STRUCTURE runs.**

| Parameter set | Run name | K | Ln Likelihood | Ln P(D) | Var[LnP(D)] |
| --- | --- | --- | --- | --- | --- |
| Lambda | Lambda_run_9 | 1 | -15226 | 192.2 | - |
| Lambda | Lambda_run_8 | 1 | -15225.1 | 190.5 | - |
| Lambda | Lambda_run_7 | 1 | -15225.3 | 191.1 | - |
| Lambda | Lambda_run_6 | 1 | -15226.1 | 192.7 | - |
| Lambda | Lambda_run_5 | 1 | -15225.4 | 191 | - |
| Lambda | Lambda_run_4 | 1 | -15225.8 | 191.9 | - |
| Lambda | Lambda_run_3 | 1 | -15226.5 | 193.2 | - |
| Lambda | Lambda_run_2 | 1 | -15226.3 | 193.1 | - |
| Lambda | Lambda_run_1 | 1 | -15225.4 | 191.1 | - |
| Lambda | Lambda_run_10 | 1 | -15225.1 | 190.5 | - |
| Lambda | Lambda_run_20 | 2 | -14984.8 | 683.3 | 2.3865 |
| Lambda | Lambda_run_19 | 2 | -14993.2 | 707.2 | 2.1987 |
| Lambda | Lambda_run_18 | 2 | -14985.1 | 700.3 | 1.2266 |
| Lambda | Lambda_run_17 | 2 | -15031.9 | 775 | 2.9983 |
| Lambda | Lambda_run_16 | 2 | -15587.3 | 1802.9 | 1.0433 |
| Lambda | Lambda_run_15 | 2 | -15019.1 | 748.9 | 2.7975 |
| Lambda | Lambda_run_14 | 2 | -15235.7 | 1042.1 | 1.5242 |
| Lambda | Lambda_run_13 | 2 | -15119.8 | 810.2 | 3.2896 |
| Lambda | Lambda_run_12 | 2 | -15069 | 851.4 | 2.0064 |
| Lambda | Lambda_run_11 | 2 | -15336.6 | 1383.7 | 1.9847 |
| Lambda | Lambda_run_30 | 3 | -14837.6 | 1156.1 | 1.3695 |
| Lambda | Lambda_run_29 | 3 | -14846.3 | 1166.6 | 1.4134 |
| Lambda | Lambda_run_28 | 3 | -14785.5 | 959.6 | 0.9464 |
| Lambda | Lambda_run_27 | 3 | -14751.3 | 866.6 | 2.9508 |
| Lambda | Lambda_run_26 | 3 | -14901.7 | 1151.5 | 2.6513 |
| Lambda | Lambda_run_25 | 3 | -15113.4 | 1666.3 | 0.8676 |
| Lambda | Lambda_run_24 | 3 | -14793.7 | 960 | 1.0507 |
| Lambda | Lambda_run_23 | 3 | -14765.6 | 924.9 | 1.5865 |
| Lambda | Lambda_run_22 | 3 | -14739.9 | 856.8 | 2.2441 |
| Lambda | Lambda_run_21 | 3 | -14728.9 | 809.7 | 3.1725 |
| Lambda | Lambda_run_40 | 4 | -14756.9 | 1490 | 3.0164 |
| Lambda | Lambda_run_39 | 4 | -14576.4 | 1185.3 | 1.3126 |
| Lambda | Lambda_run_38 | 4 | -14568.2 | 1188 | 1.1283 |
| Lambda | Lambda_run_37 | 4 | -14532.9 | 1117.5 | 1.1798 |
| Lambda | Lambda_run_36 | 4 | -14535.3 | 1110.9 | 1.2454 |
| Lambda | Lambda_run_35 | 4 | -14593.6 | 1211.8 | 1.2973 |
| Lambda | Lambda_run_34 | 4 | -14623.3 | 1276.8 | 1.2694 |
| Lambda | Lambda_run_33 | 4 | -14638 | 1291.1 | 1.4534 |
| Lambda | Lambda_run_32 | 4 | -14524.9 | 1097.6 | 1.3863 |
| Lambda | Lambda_run_31 | 4 | -14659.1 | 1336.3 | 1.858 |
| Lambda | Lambda_run_50 | 5 | -14509.8 | 1626.5 | 0.8562 |
| Lambda | Lambda_run_49 | 5 | -14504.5 | 1612.2 | 0.6347 |
| Lambda | Lambda_run_48 | 5 | -14394.7 | 1424.5 | 1.0572 |
| Lambda | Lambda_run_47 | 5 | -14540 | 1697.5 | 0.6893 |
| Lambda | Lambda_run_46 | 5 | -14507.1 | 1617 | 0.7937 |
| Lambda | Lambda_run_45 | 5 | -14437.3 | 1489.8 | 0.7491 |
| Lambda | Lambda_run_44 | 5 | -14564.6 | 1741.7 | 1.1521 |
| Lambda | Lambda_run_43 | 5 | -14469.7 | 1546.8 | 0.9252 |
| Lambda | Lambda_run_42 | 5 | -14420.9 | 1479.8 | 0.6041 |
| Lambda | Lambda_run_41 | 5 | -14722.7 | 2042.2 | 0.6675 |
| Lambda | Lambda_run_61 | 6 | -14738.6 | 2506.3 | 1.1139 |
| Lambda | Lambda_run_60 | 6 | -14420.6 | 1899.8 | 0.7056 |
| Lambda | Lambda_run_59 | 6 | -14456.5 | 1960 | 0.5926 |
| Lambda | Lambda_run_58 | 6 | -14459 | 1999.1 | 0.4529 |
| Lambda | Lambda_run_57 | 6 | -14431.9 | 1962.8 | 0.6642 |
| Lambda | Lambda_run_56 | 6 | -14330 | 1751.2 | 0.439 |
| Lambda | Lambda_run_55 | 6 | -14426.4 | 1920.7 | 0.2891 |
| Lambda | Lambda_run_54 | 6 | -14655.4 | 2383.1 | 0.3542 |
| Lambda | Lambda_run_53 | 6 | -14425.9 | 1937.3 | 0.3329 |
| Lambda | Lambda_run_52 | 6 | -14420.4 | 1900.6 | 0.6508 |
| Lambda | Lambda_run_51 | 6 | -14380.7 | 1836.7 | 0.4938 |


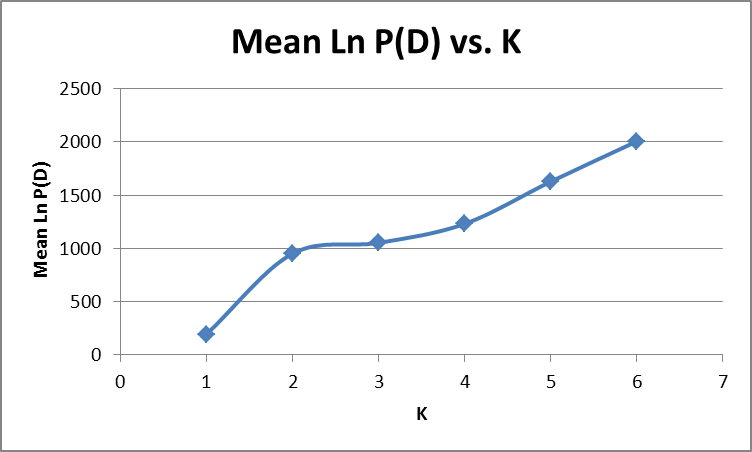


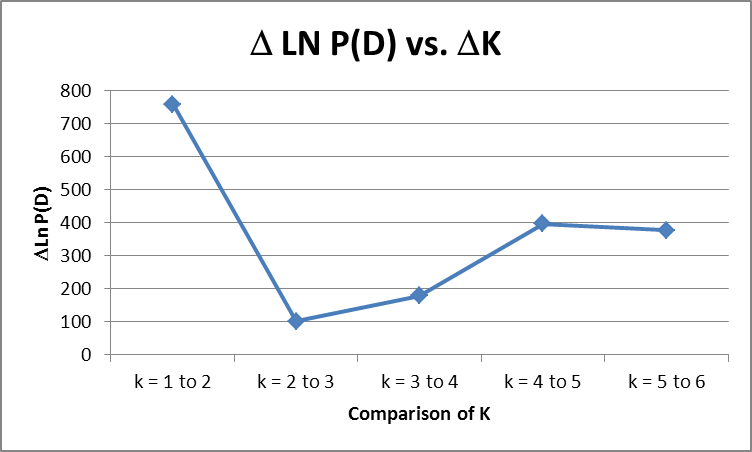

Supplement: Supplementary file 1 [file ece30001-0026-SD1.doc]
